# The Cyclic AMP Cascade Is Altered in the Fragile X Nervous System

**DOI:** 10.1371/journal.pone.0000931

**Published:** 2007-09-26

**Authors:** Daniel J. Kelley, Richard J. Davidson, Jamie L. Elliott, Garet P. Lahvis, Jerry C. P. Yin, Anita Bhattacharyya

**Affiliations:** 1 Waisman Laboratory for Brain Imaging and Behavior, Waisman Center, University of Wisconsin, Madison, Wisconsin, United States of America; 2 Neuroscience Training Program, University of Wisconsin School of Medicine and Public Health, University of Wisconsin, Madison, Wisconsin, United States of America; 3 Medical Scientist Training Program, University of Wisconsin School of Medicine and Public Health, University of Wisconsin, Madison, Wisconsin, United States of America; 4 Department of Surgery, University of Wisconsin, Madison, Wisconsin, United States of America; 5 Department of Genetics, University of Wisconsin, Madison, Wisconsin, United States of America; 6 Stem Cells and Developmental Disorders Laboratory, Waisman Center, University of Wisconsin, Madison, Wisconsin, United States of America; National Institutes of Health, United States of America

## Abstract

Fragile X syndrome (FX), the most common heritable cause of mental retardation and autism, is a developmental disorder characterized by physical, cognitive, and behavioral deficits. FX results from a trinucleotide expansion mutation in the fmr1 gene that reduces levels of fragile X mental retardation protein (FMRP). Although research efforts have focused on FMRP's impact on mGluR signaling, how the loss of FMRP leads to the individual symptoms of FX is not known. Previous studies on human FX blood cells revealed alterations in the cyclic adenosine 3′, 5′-monophosphate (cAMP) cascade. We tested the hypothesis that cAMP signaling is altered in the FX nervous system using three different model systems. Induced levels of cAMP in platelets and in brains of fmr1 knockout mice are substantially reduced. Cyclic AMP induction is also significantly reduced in human FX neural cells. Furthermore, cAMP production is decreased in the heads of FX Drosophila and this defect can be rescued by reintroduction of the dfmr gene. Our results indicate that a robust defect in cAMP production in FX is conserved across species and suggest that cAMP metabolism may serve as a useful biomarker in the human disease population. Reduced cAMP induction has implications for the underlying causes of FX and autism spectrum disorders. Pharmacological agents known to modulate the cAMP cascade may be therapeutic in FX patients and can be tested in these models, thus supplementing current efforts centered on mGluR signaling.

## Introduction

Fragile X syndrome (FX) is characterized by physical, cognitive, and behavioral deficits [1]. A CGG trinucleotide expansion in the fmr1 gene suppresses production of the FX mental retardation protein (FMRP), an RNA binding protein that regulates translation through interaction with dendritic ribosomes and has a role in mRNA shuttling [2]–[5]. Both mouse and Drosophila models of FX have been characterized [6], [7]. Rodent models are surprisingly mild in their phenotypes compared to the severity of the human disease. Drosophila models show stronger phenotypes with defects in brain development, axon pathfinding, and effects on courtship-based learning and memory formation and circadian rhythms. A major advance occurred when Huber and Bear reported deficits in synaptic plasticity in FX mice. Their “mGluR theory of FX” proposes that there is excessive signaling through the mGluR5 subtype of glutamatergic receptors [8]. Experiments in hippocampus show that a form of synaptic plasticity, long-term depression (LTD), can be induced with either type I mGluR (mGluR1 and mGluR5) agonists or with low frequency electrical stimulation [9]. LTD persistence requires de novo protein synthesis and, remarkably, involves FMRP. FX mice show excessive LTD, suggesting that FMRP is normally involved in a pathway terminating this type of LTD. Contemporaneously, FX mice were shown to be sensitive to audiogenic seizures, a striking phenotype similar to some FX patients who also suffer seizures [10]. Key pharmacological experiments in both models show that mGluR5 antagonists can reverse most, but not all, of the endophenotypes [8], [11], [12]. It is therefore possible that there are other defects within the FX brain that are not reversible when mGluR signaling is inhibited.

In the early 1990s, Berry-Kravis and colleagues postulated that another intracellular signaling pathway, cyclic AMP (cAMP), contributes to FX neuropathophysiology [13]. Cyclic AMP is produced by adenylate cyclase (AC), degraded by phosphodiesterase (PDE), and can bind PKA, GEFs, and CNG channels. AC can be stimulated by G proteins or forskolin and modulated by Ca^2+^, G proteins, PKA, PKC, and CaMK [14]. The cAMP system is critical in many aspects of neural development [15] and may be important in autism susceptibility [16], [17]. Cyclic AMP production is decreased in non-neural cells from FX patients and this alteration is FMRP dependent [18], [19]. This “cAMP theory of FX” posits that alterations in the cAMP pathway may result from dysregulated expression of proteins involved in the cAMP cascade either (1) in parallel with the mGluR theory using a mechanism independent of mGluR or (2) in series with the mGluR theory through a mechanism downstream of mGluR overactivity.

The variable clinical presentation of FX and the partial reversal of phenotypes achievable using mGluR antagonists suggest that excessive mGluR signaling is difficult to correct within the mGluR pathway or that parallel pathways are involved. Therefore, we investigated the cAMP theory in the FX central nervous system. We tested whether the cAMP defect is conserved across species, thereby indicating a common mechanism for the cAMP defect. If FMRP loss limits cAMP production in the FX brain *in vivo*, pharmacologic agents known to act through the cAMP cascade may be therapeutic to FX patients.

## Results

**Figure 1:**
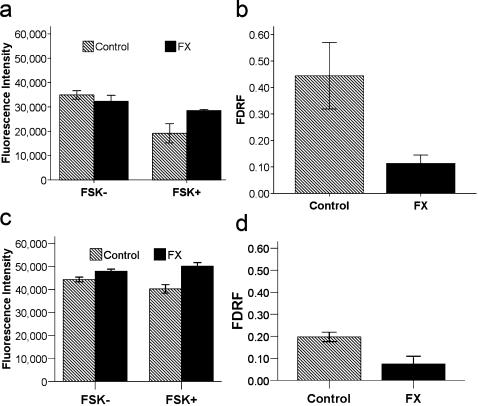
cAMP production is decreased in FX mice. Platelets (Control n = 3, FX n = 5) and cortical membranes (n = 3 for each group) from wildtype and fmr1 knockout mice were stimulated with forskolin and levels of cAMP were measured (each n averaged in triplicate). Raw fluorescence (RF) is an inverse index of cAMP levels. The change in cAMP production was assessed using the fractional decrease in RF (FDRF) = (No Forskolin−Forskolin)/(No Forskolin). (a) Mouse platelet RF is comparable in the absence of forskolin [t(6) = 0.99; p = 0.36] but significantly different between groups (GroupxStimulus:[F(1,12) = 7.56;p = 0.018]) in the presence of forskolin [t(6) = −2.62; p = 0.040]. (b) Mouse platelet FDRF shows reduced cAMP induction in FX platelets [t(6) =  3.46; p = 0.014]. (c) Mouse cortex RF is comparable between groups in the absence [t(4) = 1.22;p = 0.29] or presence [t(4) = −1.92;p = 0.13] of forskolin. (d) Mouse cortex FDRF shows reduced FX cAMP induction [t(4) = 3.01; p = 0.04]. Error bars are SEM.

To test the hypothesis that there is a highly conserved relationship between FMRP and cAMP activity, we examined cAMP production in mouse, fly and human systems containing a non-functional fmr1 gene. Forskolin is a general activator of adenylate cyclases. Berry-Kravis and Sklena reported that normal human platelets show a greater forskolin-mediated increase in cAMP than platelets from individuals with FX [20]. We investigated this effect in wild-type and fmr1 knockout mouse platelets using a fluorescence-based assay, where raw fluorescence (RF) decreases when cAMP levels increase. Figure 1a shows that untreated platelets have similar levels of RF, but forskolin treated wild-type platelets show a greater decrease than comparably treated fmr1 knockout platelets. When the fractional decrease in RF (FDRF) is calculated, fmr1 knockout mouse platelets show less of a decrease (indicating reduced cAMP induction) compared to wild type platelets [t(6) =  3.46; p = 0.014] (Figure 1b).

To extend these results to brain, we measured cAMP levels from wild-type and fmr1 knockout mouse cortical membranes. Although the RF was comparable between groups in the absence [t(4) = 1.22; p = 0.29] or presence [t(4) = −1.92; p = 0.13] of forskolin (Figure 1c), the FDRF is significantly less in fmr1 knockout tissue than wild-type tissue, indicating that the FX cortical tissue shows reduced cAMP induction [t(4) = 3.01; p = 0.04] (Figure 1d). To determine whether the cAMP defect is a ubiquitous feature of the FX knockout mouse phenotype, cAMP responses to forskolin from platelets and cortex from wild-type and fmr1 knockout mice were compared as a contrast within a three way ANOVA (GroupxStimulusxOrganSystem). The fmr1 knockout brain and blood consistently produce less cAMP than wild-type tissue based on RF measures [GroupxStimulus: F(1,20) = 10.50; p = 0.004] and FDRF [F(1,10) = 14.92; p = 0.003].

**Figure 2:**
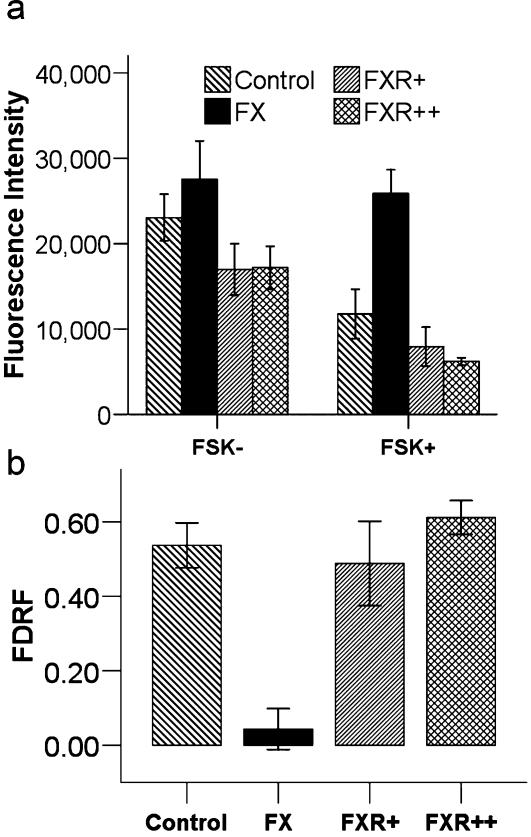
cAMP production is decreased in FX fly. Head membranes from wildtype, FX, FXR+, FXR++ flies were stimulated with forskolin and levels of cAMP were measured. Raw fluorescence (RF) is an inverse index of cAMP levels. The change in cAMP production was assessed using the fractional decrease in RF (FDRF) = (No Forskolin−Forskolin)/(No Forskolin). (a) RF-based cAMP measures in the fly head (Control n = 10, FX n = 3, FXR+ n = 6; FXR++ n = 6 sets of 20 heads) are comparable between groups in the absence of forskolin [F(3,21) = 1.96; p = 0.15], but not its presence [F(3,21) = 6.23;p = 0.003], since RF intensity in FX flies is larger than the others [t(21) = −4.10;p = 0.000]. (b) Fly head FDRF shows reduced FX cAMP induction relative to control membranes [FDRF:t(21) = 3.85;p = 0.001] and to the FX flies rescued with a single or multiple transgenes [FXR+FDRF:t(21) = 3.23;p = 0.004; FXR++FDRF:t(21) = 4.13;p = 0.000]. Error bars are SEM.

Using the Drosophila model, we varied the gene dosage of the fly FX gene (dfmr1) (0 = FX, 1 = FXR+, 2 = FXR++), and measured cAMP production from head membrane preparations in the presence or absence of forskolin. RF-based cAMP measures are comparable among wild-type, FX, FXR+, and FXR++ flies in the absence of forskolin [F(3,21) = 1.96; p = 0.15]. However, in the presence of forskolin [F(3,21) = 6.23; p = 0.003], FX flies have a larger RF compared to all other groups taken collectively, [t(21) = −4.1; p = 0.000], or individually (wild-type [t(21) = −3.16; p = 0.005], FXR+[t(21) =  −3.74; p = 0.001], and FXR++ [t(21) = −4.10; p = 0.001]). In the presence of forskolin, the wild-type flies are indistinguishable from the rescue flies [t(21) = 1.61; p = 0.123] with a single [FXR+ FDRF:t(21) = 1.09; p = 0.29] or multiple transgenes [FXR++ FDRF:t(21) = 1.58; p = 0.13] (Figure 2a). cAMP production, as assessed using FDRF, is comparable among wild-type and the group of rescued flies [FDRF:t(21) = −0.155; p = 0.88] with one [FXR+ FDRF:t(21) = 0.485; p = 0.632] or two transgenes [FXR++ FDRF:t(21) = −0.74; p = 0.46]. However, FDRF and thus cAMP induction, is diminished in FX relative to wild-type membranes [FDRF:t(21) = 3.85; p = 0.001], or relative to the group of rescued FX flies [FDRF:t(21) = 4.03; p = 0.001] with a single [FXR+ FDRF:t(21) = 3.23; p = 0.004] or multiple transgenes [FXR++ FDRF:t(21) = 4.13; p = 0.000] (Figure 2b). Therefore, FX mutant flies show less of an induction in cAMP in response to forskolin, and either one or two rescue transgenes alleviate this effect.

**Figure 3:**
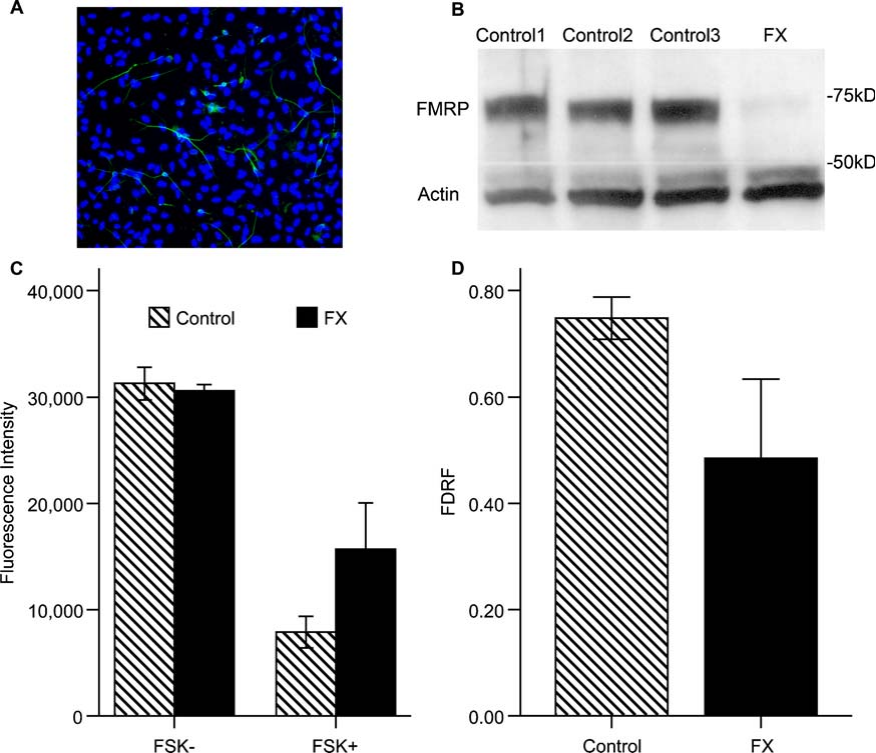
cAMP production is decreased in human FX neural cells. Unaffected and FX human neural cells were stimulated with forskolin and levels of cAMP were measured. Raw fluorescence (RF) is an inverse index of cAMP levels. The change in cAMP production was assessed using the fractional decrease in RF (FDRF) = (No Forskolin−Forskolin)/(No Forskolin). (a) Neurons (MAP2, green) are present among cells (Hoechst stain, nuclei in blue) in 9 week FX cultures. (b) Extracts of human neural cells were immunoblotted with antibodies to FMRP and actin. FX cells (FX) have reduced levels of FMRP compared to 3 controls (control 1, 2, 3). Similar levels of actin are detected in each sample. (c) Human neural cell RF (2 Controls, each with n = 3; FX, n = 3) is comparable between groups in forskolin's absence [t(6) = 0.82; p = 0.44] but FX cAMP production is lower than controls in the presence of forskolin [RF: t(6) = −3.40; p = 0.015]. (d) Human neural cell FDRF was reduced in FX [t(6) = 3.43; p = 0.01]. Error bars are SEM for FX and pooled SEM for controls. (Pooled SEM)^2^ = SEM_1_^2^+SEM_2_^2^−2*SEM_1_*SEM_2_.

To extend these experiments to human cells, we examined forskolin's effects on cultures of human FX and unaffected control neural cells. Both control and FX cultures contain neurons and astrocytes (Figure 3a), but the FX cells express markedly less FMRP than control cells (Figure 3b). Levels of cAMP in FX human neural cells are comparable to control cells in the absence of forskolin [RF:t(6) = 0.82; p = 0.44] . However, when the cultures are treated with forskolin, FX cultures produce lower levels of cAMP than controls [RF:t(6) = −3.40; p = 0.015] (Figure 3c). The fractional change in cAMP production is differentially reduced in FX compared to both controls [FDRF:t(6) = 3.43; p = 0.01] (Figure 3d) indicating that the FX neural cells show reduced cAMP induction.

## Discussion

Although FX etiology is well defined, individual differences in syndrome expression and symptoms complicate current options for treatment. To define therapeutic pathways to target, it is important to identify cellular processes affected in the FX brain. Based on previous work, we compared the inducibility of cAMP across neural models and found that cAMP induction is decreased in the FX fly, mouse and human nervous system.

The alterations we detect in FX nervous tissue have broad implications, since cAMP signaling has widespread cellular impact, potentially affecting channel function, signal transduction (GEFs and EPAC) and gene expression (CREB) [15]. The conservation of the cAMP defect in fly, mouse and human species suggests that cAMP metabolism may serve as a useful biomarker in the human disease population, and may account for some of the differences in clinical endophenotypes. An important corollary is that increasing cAMP levels could improve neuronal function and behavior in FX patients. Precedent exists for pharmacological rescue of FX behavior, since lithium or mGluR5 antagonists improve behavior in Drosophila memory formation [12]. However, it is not known if these treatments affect cAMP inducibility, or otherwise target the cAMP pathway. The prospect of using PDE4 inhibitors, such as rolipram or HT0712, is particularly appealing, since memory enhancement through facilitation of cAMP signaling may be possible without changing basal cAMP levels [21], [22]. There are numerous candidate mechanisms that might contribute to cAMP rescue of FX neural defects, including different effects on cAMP signaling, or cross talk with the MAPK, PKC, and/or IP3/Ca^2+^ pathways.

How does a decrease in FMRP lead to alterations in cAMP? FMRP is an mRNA binding protein that shuttles subsets of mRNA between the nucleus and subcellular compartments such as neuronal dendrites. Gene expression analyses in FX models suggest that the mRNAs for adenylate cyclase types 3, 5 and 6 might be decreased in FX tissue [23]–[25]. The decrease in steady-state concentrations of other candidate mRNAs could also contribute to the alterations in cAMP metabolism that we observe.

In support of the cAMP theory, our results identify cAMP alterations in the FX nervous system that may account for some of the neuropathophysiology of FX. Several lines of evidence support the view that mGluR and cAMP mediated pathways functionally interact in platelets [26], lymphocytes [27], neurons [28]–[32] and astrocytes [33]. However, further studies are required to clarify the series or parallel relationship between the cAMP and mGluR defects in FX. For example, rescue of cAMP deficits in FX with mGluR inhibitors would suggest that the pathways operate in series, with the cAMP defect presumably downstream of excessive mGluR activity. Lack of, or incomplete rescue, would support a parallel mechanism where proteins that are directly involved in, or that interact with, the cAMP cascade are dysregulated by FMRP loss either through basal or non-mGluR mechanisms. The specific mechanism of FMRP interaction with the cAMP cascade and the conserved upstream, downstream, and behavioral consequences of reduced cAMP induction can be studied within and across FX mouse, fly, and human models. Most importantly, this work lays the groundwork for future therapeutic options on FX, thus complementing current approaches focused on the mGluR pathway.

## Materials and Methods

### Mouse Platelet Membrane Preparation

Whole mouse blood (∼0.75 ml/mouse) was collected from the first systolic ejection upon decapitation and collected in EDTA solution (1mg EDTA/ml whole blood). Within 2 hours of collection, blood samples were centrifuged at 700×g for 10 minutes at room temperature [34]. The upper platelet-rich plasma layer (UPRP) was transferred to a fresh centrifuge tube and centrifuged at 700×g for 10 minutes at room temperature. The UPRP plasma was centrifuged at 2800×g for 15 minutes at room temperature. The platelet pellet was stored at –70°C until assayed. Pellets were thawed on ice and centrifuged at 17000×g in a microfuge for 10 minutes. Protein levels were determined with the Lowry protein assay (Bio-Rad Laboratories, Hercules, CA). 100ug protein was incubated in Krebs-Ringer Bicarbonate buffer (KRB) with 3-isobutyl-1methylxanthine (IBMX; a non-selective PDE inhibitor) at 37°C for 20 minutes in the presence of ATP plus forskolin (5 µM; solubilized in DMSO; Sigma, St. Louis, MO) or DMSO alone and then assayed for cAMP.

### Mouse Brain Cortical Membrane Preparation

Cerebral cortices were harvested from three-month old wild type and age-matched FX (fmr1 knockout) male C57/Bl6 mice. Tissue was homogenized in 25 mM HEPES, 2 mM EGTA, pH 7.4, and centrifuged at 750×g for 10 minutes at 4°C. The supernatant was centrifuged at 45,000×g for 60 minutes at 4°C [35]. Pellets were resuspended in HEPES buffer and stored at –80°C until assayed. Cortical membranes were thawed and centrifuged at 17000×g in a microfuge for 10 minutes at 4°C. The pellet was resuspended in 1X KRB-IBMX solution. 300ug of protein was incubated in KRB-IBMX at 37°C for 20 minutes in the presence of ATP plus forskolin or DMSO and then assayed for cAMP.

### Drosophila Head Membrane Preparation

Wild-type, FX, FX plus a dfmr rescue transgene (FXR+), and FX plus a double dfmr transgene (FXR++) male Drosophila Melanogaster were frozen, heads isolated, and stored at −70°C [36]. Fifty heads were homogenized with a motorized pellet pestle (Kontes; Vineland, NJ) in 100 uL ice-cold homogenization buffer (15 mM HEPES pH 7.5, 10mM KCl, 5 mM MgCl_2_, 0.1 mM EDTA, 0.1 mM EGTA, 5% sucrose, Roche Complete Protease Inhibitor cocktail tablet). The suspension was allowed to settle 5 min on ice to remove the chitin exoskeleton. To remove eye pigment, 5mg acid rinsed charcoal was added [37] and the suspension was centrifuged three times at 200×g for 2 minutes to remove the charcoal. The supernatant was centrifuged at 14,000×g for 15 minutes and resuspended in 50 uL of 1X KRB-IBMX. 2 ug Drosophila head membranes was incubated in KRB-IBMX at 37°C for 20 minutes in the presence of ATP plus forskolin or DMSO and then assayed for cAMP.

### Human Neural Cells

Human FX and unaffected neural cells were differentiated from FX and unaffected human neural progenitor cells (hNPCs). Human neural progenitor cells were derived from post-mortem cerebral cortices of 3 control fetuses (gestational ages 81–98 days) and a full mutation male Fragile X fetus (gestational age approximately 98 days) [38]. Human fetal tissue was obtained according to the guidelines recommended by National Institutes of Health for the collection of fetal tissue and set out by the University of Wisconsin-Madison. Institutional Review Board approval was obtained for all of these studies. FX and control hNPCs were plated onto laminin/poly-L-lysine flasks in DMEM/Ham's F12 media (Invitrogen, Carlsbad CA ) with penicillin, streptomycin, amphotericin B (PSA, 1%; Invitrogen, Carlsbad CA) and supplemented with B27 (2%; Invitrogen, Carlsbad CA) and NT4 (20 ng/ml; Peprotech) for 7 days [39]. Cells were then dissociated and grown at a density of 5000 cells/ul for 9–12 weeks in Neurobasal media (Invitrogen, Carlsbad CA ) with penicillin, streptomycin, amphotericin B (PSA, 1%; Invitrogen, Carlsbad CA) and glutamine (Sigma, St. Louis, MO) and supplemented with fetal calf serum (10%; Invitrogen, Carlsbad CA), B27 (2%; Invitrogen, Carlsbad CA) and NT4 (20ng/ml; Peprotech). For cAMP assays, cells were enzymatically dissociated and suspended to a final concentration of 5×10^5^ cells/ml in KRB-IBMX buffer. After 15 minutes, cells were stimulated with 50 uM forskolin or DMSO alone for 30 minutes at room temperature and assayed for cAMP levels.

#### Immunocytochemistry

Cultures were fixed in 4% paraformaldehyde and washed in PBS. Fixed cells were blocked and permeabilized in 5% goat serum with 0.2% Triton X-100 and subsequently incubated with an antibody to MAP2 (1∶1000; Sigma, St. Louis, MO) followed by incubation with a fluorescent secondary antibody. Hoechst 33258 (Sigma, St. Louis, MO) was used as a nuclear stain.

#### Western blots

FX and control neural cells were harvested in lysis buffer (20 mM Tris, pH 8, 137 mM NaCl, 1% NP-40, 10% glycerol, protease inhibitors) and cleared by centrifugation. Protein extracts (25 ug) and molecular weight standards were size fractionated with SDS-PAGE and immunoblotted with antibodies to FMRP (Chemicon/Millipore, Billerica, MA) and actin (Sigma, St. Louis, MO). Proteins were visualized by incubation with horseradish peroxidase-coupled secondary antibodies and enhanced chemiluminescence (Amersham Biosciences), followed by exposure onto x-ray film.

### Flourescent cAMP Assay

The Bridge-It cAMP designer fluorescence assay system (Mediomics, LLC, St. Louis, MO) was used to measure cyclic AMP levels in all samples [40]. cAMP is quantified as a reduction in fluorescence when CAP (cAMP Receptor Protein), a bacterial transcription factor whose DNA binding affinity is high in the presence of cAMP and low in cAMP's absence, binds to DNA fluorescently tagged at the CAP binding site. Binding quenches fluorescence. This fluorescence assay has a 5 nM cAMP detection level and was carried out according to manufacturer's specifications. Fluorescence intensity was evaluated by using a Safire II plate reader (Tecan, Inc., St. Louis, MO). Raw fluorescence (RF) was used as an index of static cAMP levels at rest and after stimulation. The change in cAMP production was assessed using the fractional decrease in RF (FDRF) = (No Forskolin−Forskolin)/(No Forskolin), a statistic that measures the percentage of fluorescence quenching and is both reproducible for a given cAMP level and independent of the instrument used to measure fluorescence.

Statistical analyses were conducted in SPSS 14.0 (SPSS, Inc, Chicago, IL) and evaluated for two-tailed significance. All fluorescence trials were replicated in triplicate unless otherwise noted. Human cell studies were blinded with respect to FX status and unblinded for analysis.
